# *Tityus stigmurus*-Venom-Loaded Cross-Linked Chitosan Nanoparticles Improve Antimicrobial Activity

**DOI:** 10.3390/ijms25189893

**Published:** 2024-09-13

**Authors:** Fiamma Gláucia-Silva, João Vicente Pereira Torres, Manoela Torres-Rêgo, Alessandra Daniele-Silva, Allanny Alves Furtado, Sarah de Sousa Ferreira, Guilherme Maranhão Chaves, Francisco Humberto Xavier-Júnior, Karla Samara Rocha Soares, Arnóbio Antônio da Silva-Júnior, Matheus de Freitas Fernandes-Pedrosa

**Affiliations:** 1 Laboratory of Technology and Pharmaceutical Biotechnology (Tecbiofar), Faculty of Pharmacy, Federal University of Rio Grande do Norte, General Gustavo Cordeiro de Farias Avenue, S/N, Petrópolis, Natal 59012-570, Brazil; fiammaglaucia@ufrn.edu.br (F.G.-S.); jvcnt@hotmail.com (J.V.P.T.); manoela.torres.072@ufrn.edu.br (M.T.-R.); alessandra.daniele@outlook.com (A.D.-S.); allannyfurtado@ufrn.edu.br (A.A.F.); sarahferreira@ufrn.edu.br (S.d.S.F.); karllasamara@yahoo.com.br (K.S.R.S.); arnobiosilva@gmail.com (A.A.d.S.-J.); 2 Graduate Program of Chemistry, Chemistry Institute, Federal University of Rio Grande do Norte, Senador Salgado Filho Avenue, 3000, Lagoa Nova, Natal 59012-570, Brazil; 3 Laboratory of Medical and Molecular Micology, Department of Clinical and Toxicological Analyses, Faculty of Pharmacy, Federal University of Rio Grande do Norte, General Gustavo Cordeiro de Farias Avenue, S/N, Petrópolis, Natal 59012-570, Brazil; guilherme.chaves@ufrn.br; 4 Laboratory of Pharmaceutical Biotechnology (BioTecFarm), Department of Pharmaceutical Sciences, Federal University of Paraiba, Campus Universitário I, Castelo Branco III, Cidade Universitária, João Pessoa 58051-900, Brazil; fhxj@academico.ufpb.br

**Keywords:** antimicrobial, biodegradable nanocarrier, chitosan, nanoparticle, *Tityus stigmurus* scorpion

## Abstract

The rapid resistance developed by pathogenic microorganisms against the current antimicrobial pool represents a serious global public health problem, leading to the search for new antibiotic agents. The scorpion *Tityus stigmurus*, an abundant species in Northeastern Brazil, presents a rich arsenal of bioactive molecules in its venom, with high potential for biotechnological applications. However, venom cytotoxicity constitutes a barrier to the therapeutic application of its different components. The objective of this study was to produce *T. stigmurus*-venom-loaded cross-linked chitosan nanoparticles (Tsv/CN) at concentrations of 0.5% and 1.0% to improve their biological antimicrobial activity. Polymeric nanoparticles were formed with a homogeneous particle size and spherical shape. Experimental formulation parameters were verified in relation to mean size (<180 nm), zeta potential, polydispersity index and encapsulation efficiency (>78%). Tsv/CN 1.0% demonstrated an ability to increase the antimicrobial venom effect against *Staphylococcus aureus* bacteria, exhibiting an MIC value of 44.6 μg/mL. It also inhibited different yeast species of the *Candida* genus, and Tsv/CN 0.5% and 1.0% led to a greater inhibitory effect of *C. tropicalis* and *C. parapsilosis* strains, presenting MIC values between 22.2 and 5.5 µg/mL, respectively. These data demonstrate the biotechnological potential of these nanosystems to obtain a new therapeutic agent with potential antimicrobial activity.

## 1. Introduction

Antibiotic resistance can be triggered by the misuse or over-prescription of antibiotics and has become a serious global public health problem that threatens patient care, healthcare systems and the world economy [1,2,3]. Bacterial infections caused by multidrug resistant strains are among the important causes of death around the world [4,5,6]. Currently, approximately 700,000 deaths occur annually in the world due to drug-resistant diseases. According to the World Health Organization (WHO), if no action is taken, that number could increase to 10 million deaths globally per year by 2050 [7]. Furthermore, the decline in the development of new antimicrobials by the pharmaceutical industry aggravates this serious health problem, leading to an urgent need to search for new therapeutic alternatives to combat antimicrobial resistance [8,9].

Studies with animal venom are relevant, mainly due to the presence of components with a high biotechnological and medicinal potential. Many toxin components have served as templates or as prototypes for developing innovative pharmaceutical drugs employed in treating several human diseases, including diabetes, stroke, heart attack and cancer, among other clinically relevant pathologies [10]. Scorpion venom is a rich source of pharmacologically active compounds, and much attention has been given to the presence of molecules with antimicrobial action against a variety of microorganisms [11,12,13]. Among these components, scorpion venom peptides without disulfide bridges have shown multifunctional activity, revealing antiproliferative action against different cancer cell lineages, immunomodulatory effects, as well as antibacterial, antifungal, antiviral and antiparasitic effects against different microorganisms [14]. These antimicrobial peptides (AMPs) from scorpions commonly have a positive charge and can interact with anionic phospholipids of the bacterial membrane through electrostatic interactions, killing the microorganism through pore formation, and consequently disrupting cell integrity. In addition, these peptides can recruit and activate immune system cells to act in the inflammatory process and enhance microbial elimination [13,15,16]. The AMPs can also selectively act on target cells, being recognized as innate immunity and self-cleaved components to form amino acids [17].

*Tityus stigmurus* (Thorell, 1876) (Scopiones: Buthidae) is a species of scorpion distributed in northeastern Brazil, mainly in the Caatinga region, and the state of São Paulo, Brazil [18,19,20]. Analysis of the transcriptome of the *T. stigmurus* venom (Tsv) gland showed a predominance of antimicrobial peptides (AMPs) in the total transcripts, corresponding to the most abundant component when compared to other toxins in the venom [21]. Despite the high pharmacotherapeutic potential of AMPs, their susceptibility to proteolysis, high production costs, in vivo toxicity and hemolytic and cytotoxic effects limit their therapeutic applicability [8]. Therefore, pharmaceutical applications have been sought as a useful approach for the potential use of these molecules as pharmacological agents.

In turn, nanotechnology comes with promising solutions for major problems in various fields of science and technology, and has proven to be useful in developing new therapies to combat multidrug-resistant microbial strains [9]. Nanocarriers have been used to increase the therapeutic efficacy of proteins and peptides, since they can minimize side-effects, control the pharmacokinetic profile, send immune system cells to the target organ, reduce toxicity and protect the molecules from degradation [16,22,23]. Chitosan is a natural copolymer which has been used to develop nanoparticles with biotechnological potential [24,25,26,27]. The main properties of chitosan are biodegradability and biocompatibility, and it is a non-antigenic and non-toxic polymer [28,29].

In view of the above, this study aimed to evaluate the antimicrobial potential of *T. stigmurus*-venom-loaded cross-linked chitosan nanoparticles (Tsv/CN) at concentrations of 0.5% and 1.0%. The nanocarriers were produced and analyzed regarding their physicochemical characteristics and in vitro antibacterial and antifungal activity.

## 2. Results

### 2.1. Preparation and Physicochemical Characterization of Tityus stigmurus-Venom-Loaded Cross-Linked Chitosan Nanoparticles

The physicochemical characterization of chitosan nanoparticles (CN) and *Tityus stigmurus*-venom-loaded cross-linked chitosan nanoparticles at concentrations of 0.5% (Tsv/CN 0.5%) and 1.0% (Tsv/CN 1.0%) revealed homogeneous particle sizes of 176 nm and 106 nm, with a polydispersity index (PdI) around 0.5 for both nanosystems, cationic charge and encapsulation efficiency higher than 80% and 78% for Tsv/CN 0.5% and Tsv/CN 1.0%, respectively (Table 1).

**Figure 1:**
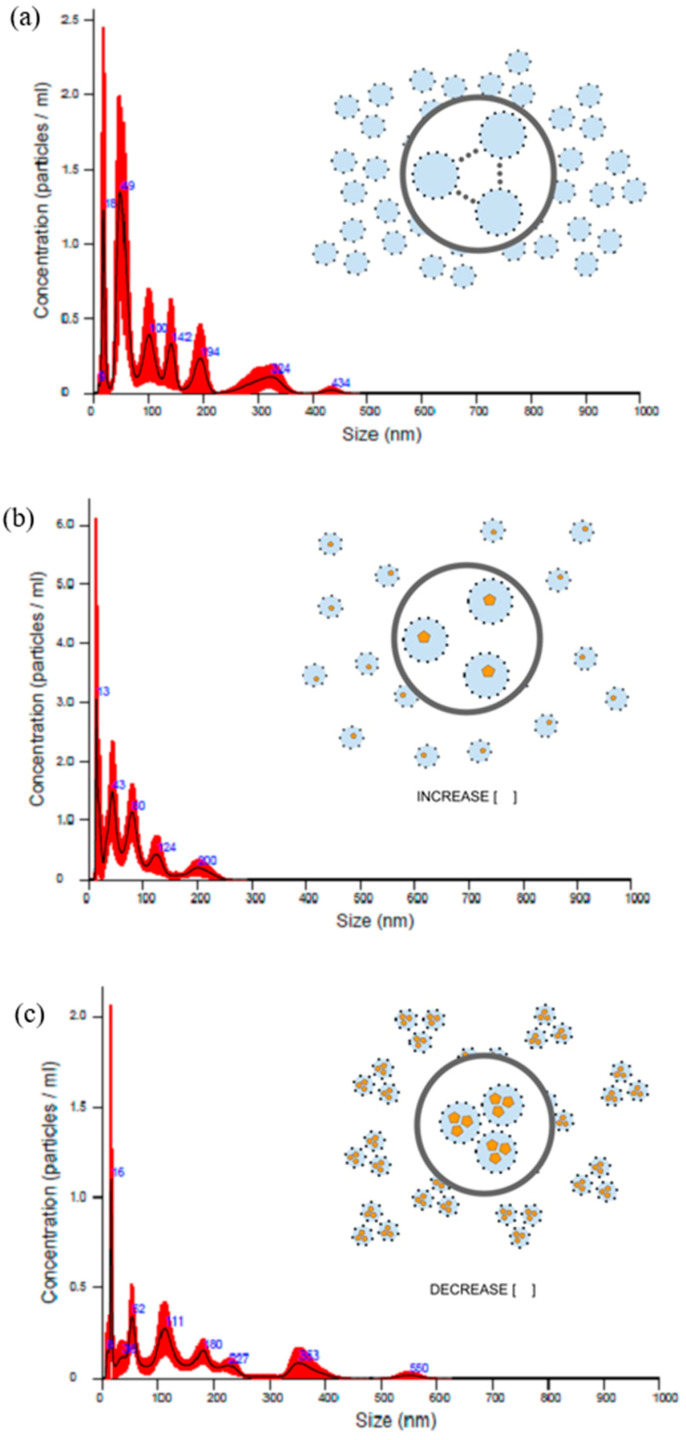
Nanoparticle tracking analyzer of (**a**) chitosan nanoparticles and *Tityus stigmurus*-venom-loaded cross-linked chitosan nanoparticles at concentrations of (**b**) 0.5% and (**c**) 1.0%.

Next, the nanoparticle dispersion was analyzed through tracking individual particles, and it was possible to observe that the particle size was smaller than 200 nm, corroborating the data obtained through dynamic light scattering (DLS). Analysis scans of the nanoparticle tracking analyzer (NTA) verify individual particles and capture several frames of the solution and then correlate the particle size with the particle concentration in the solution, so a small size difference between the results obtained through the NTA and DLS analysis is acceptable. It is also possible to identify the concentration of nanoparticles present per mL of nanoparticle solution, which was 6.81 × 10^7^ ± 1.58 × 10^7^ particles/mL for the CN; 1.03 × 10^8^ ± 1.16 × 10^7^ particles/mL for Tsv/CN 0.5%; and 3.86 × 10^7^ ± 4.01 × 10^6^ particles/mL for the Tsv/CN 1.0% sample. It was also possible to observe that the nanoparticle concentration tends to increase when scorpion venom at a concentration of 0.5% was applied to the nanosystem, which may suggest that the venom (at low concentrations) adsorbed on the surface of the nanoparticles contributes to the stability of particles which are compatible with each other, and this interaction between nanoparticles tends to increase when the venom concentration is increased (Figure 1).

**Figure 2:**
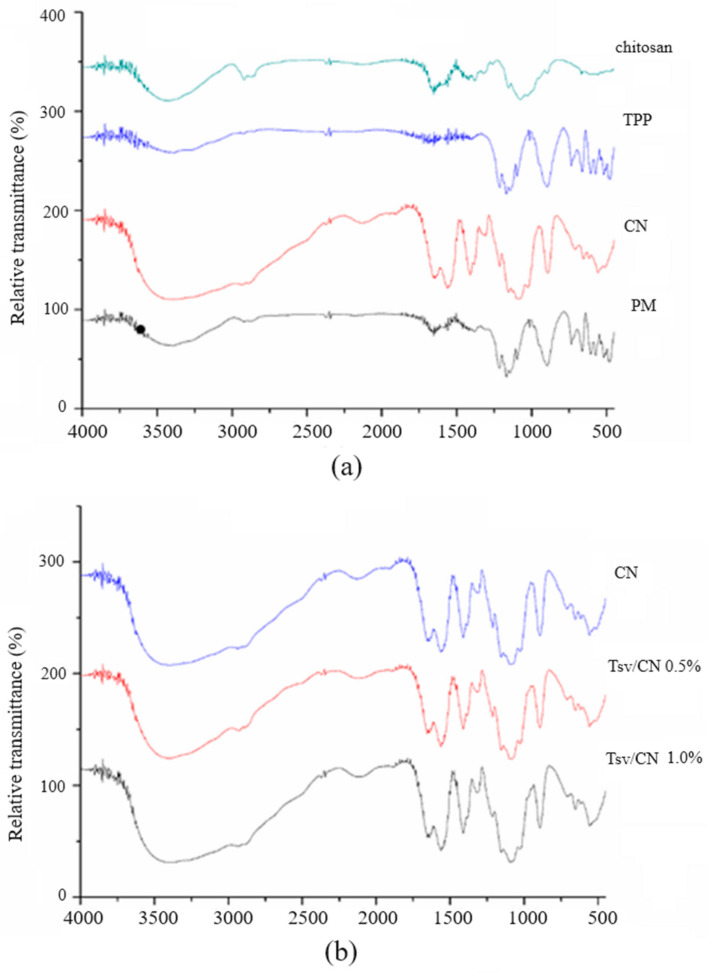
Fourier transform infrared spectroscopy of the chitosan nanoparticles and *Tityus stigmurus*-venom-loaded cross-linked chitosan nanoparticles at 0.5% and 1.0%. (**a**) FT-IR spectra of pure chitosan, pure tripolyphosphate (TPP), chitosan nanoparticles (CN) and physical mixture (PM) (chitosan and TPP), and (**b**) *Tityus stigmurus*-venom-loaded cross-linked chitosan nanoparticles (Tsv/CN) at 0.5% and 1.0%.

These interactions between nanoparticle components were monitored using relative transmittance intensity data recorded in the Fourier transforms infrared spectroscopy (FT- IR) spectra of samples. The FT-IR spectra comparisons of pure chitosan, pure TPP, chitosan nanoparticles (CN) and a physical mixture (chitosan and TPP) are shown in Figure 2a, while Figure 2b shows the FT-IR spectra comparisons between (Tsv/CN) at 0.5% and 1.0%. The N-H and C-O bands in the nanoparticle formulation spectra corresponding to the amide group of chitosan are recorded at 1561 cm^−1^ and 1649 cm^−1^, constituting the band relative to TPP recorded at 892 cm^−1^. There was a slight reduction in the intensity of the chitosan (1561 cm^−1^ and 1649 cm^−1^) and TPP (892 cm^−1^) bands in the nanosystem containing Tsv, corroborating with venom adsorption on nanoparticle surfaces. In turn, no significant differences were identified in different FT-IR spectra recorded for (Tsv/CN) at 0.5% and 1.0% using this nanoparticle venom-loading method (Figure 2b).

**Figure 3:**
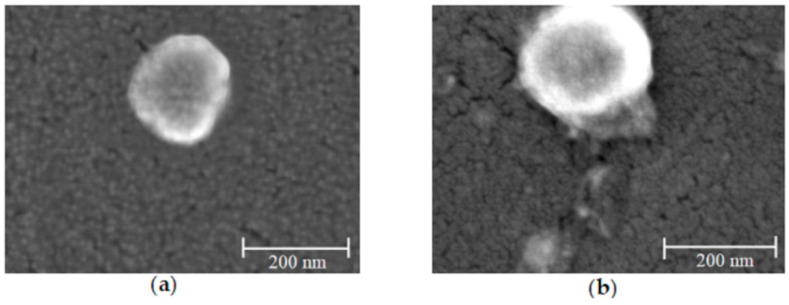
Surface of chitosan nanoparticles and *Tityus stigmurus*-venom-loaded cross-linked chitosan nanoparticles at 1.0% observed using field emission gun scanning electron microscopy. (**a**) Field emission gun scanning electron microscopy images of chitosan nanoparticles and (**b**) *Tityus stigmurus*-venom-loaded cross-linked chitosan nanoparticles at 1.0% at 200 nm.

The shape and surface of CN and Tsv/CN 1.0% were accessed by field emission gun scanning electron microscopy images (Figure 3a and Figure 3b, respectively). The images revealed apparently homogeneous and spherical nanoparticles with a smooth surface for both formations. It was also observed that venom loading did not interfere in the formation of sub 200 nm spherical nanosystems, corroborating with the particle size achieved by DLS.

### 2.2. Antibacterial and Antifungal Effect of Tityus stigmurus-Venom-Loaded Cross-Linked Chitosan Nanoparticles

The minimum inhibitory concentrations (MICs) for Tsv, CN, Tsv/CN 0.5% and Tsv/CN 1.0% were determined by the broth microdilution method [30] and are described in Table 2.

The results demonstrate that the Tsv did not present antibacterial activity for any of the tested strains (*S. aureus* and *E. coli*), even at the highest concentration (500 µg/mL). Compared to TsV, CN showed a more effective MIC for both tested strains (178.5 µg/mL), which evidenced a potential antibacterial effect for Gram-positive and Gram-negative bacteria. However, when the *T. stigmurus* venom was adsorbed in nanoparticles at concentrations of 0.5% and 1.0%, there was improved antibacterial activity for the *S. aureus* strain, demonstrating an increased dose-dependent effect for Tsv/CN 0.5% with an MIC of 89.2 µg/mL, and for Tsv/CN 1.0% of 44 µg/mL, when compared with the CN (178.5 µg/mL) and Tsv (>500 µg/mL) groups. The antibacterial activity for the *E. coli* species was not improved. 

The antifungal activity was determined against *Candida* spp. strains and the results demonstrated that the Tsv did not present antifungal activity. Nevertheless, the CN demonstrated satisfactory antifungal activity when compared with the scorpion venom. The nanoparticles loaded with *T. stigmurus* venom were able to improve antifungal activity against *C. tropicalis* and *C. parapsilosis* strains, with an MIC for Tsv/CN 0.5% of 5.5 µg/mL and Tsv/CN 1.0% of 11.1 µg/mL, respectively (Table 3).

## 3. Discussion

Many bioactive molecules have been identified in *Tityus stigmurus* scorpion venom but are still little explored in the literature [21,31]. Moreover, many bioactive components present in scorpion venom display toxic effects or suffer degradation in a physiological environment, leading to the search for different technological tools that reduce toxicity and increase stability, maintaining or improving its therapeutic potential [14,32,33].

The nanocarriers in this study were produced by the ionic gelation technique, as it is a rapid, simple, non-toxic and reproducible method able to form spherical, small and narrow-sized nanoparticles [34]. Nanoparticle formation in this method occurs from the interaction between the anionic TPP groups with amine cationic chitosan groups, inducing the cross-linking of copolymers and self-assembling of nanoparticles [25]. The ideal stoichiometry between CN and TPP to obtain stable and narrow-sized chitosan nanoparticles for protein loading were analyzed and established in previous studies developed by our research group [35].

The reduction in particle size after association with venom may be related to a competition between the anionic components of the Tsv and the phosphate group of TPP for the amine group of chitosan (cationic charge), resulting in greater adhesion and compaction of the particles. When the protein is added to the nanosystem by adsorption, in which the peptide or protein is introduced to the system after forming the nanoparticles, the negative peptide attaches to the nanoparticle surface and cannot induce change or only induces little change in the nanoparticle size.

In this study, nanoparticles were obtained with a spherical shape, smooth surface and homogeneous aspects with stable colloidal dispersions. After the nanoparticle formation, the Tsv was added into the formulation and its negative proteins anchored on the surface of particles; then, it was possible to observe a small change in the particle size after the adsorption of the venom proteins [35,36]. The shape and surface of CN and Tsv/CN were observed by field emission gun scanning electron microscopy images, and no major changes were observed after the adsorption of scorpion venom proteins. The CN revealed a high incorporation/adsorption of molecules from Tsv, showing a similar physical–chemical profile (particle size and nanoparticle morphology) effect with the multifunctional peptide TistH (*T. stigmurus* hypotensin) of *T. stigmurus* scorpion venom associated with CN, with an incorporation efficiency of 96.5 and 100% at 0.5% and 10%, respectively [36,37]; this is corroborated by previous studies in which venom from *Bothrops jararaca* and *Bothrops erythromelas* snakes and *Tityus serrulatus* scorpion venom were encapsulated in chitosan nanoparticles with a high efficiency [37,38].

The positive charge potential on the surface of the CN and its ability to interact with biomolecules such as proteins and nucleic acids [39,40,41], associated with the predominance of components with low molecular mass and anionic character in the Tsv [21], are factors which contribute to this high adsorption capacity of the venom molecules to the nanoparticle, not inducing relevant changes in the characteristics of the nanosystem. The self-assembly formation of *T. stigmurus*-venom-loaded cross-linked chitosan nanoparticles occurred due to the interactions between anionic molecules of the venom and the cationic charge of the chitosan, resulting in the lower intensity of the TPP chemical stretching (recorded at 892 cm^−1^) in formulating nanoparticles associated with the venom by FT-IR spectra. Cationic amino groups NH^3+^ from chitosan interact with anionic P=O and O-P-O from TPP, resulting in the formation of chitosan–TPP cross-linking. Previous studies have demonstrated that the decrease in the characteristic band of the TPP molecule (892 cm^−1^) after association with the venom components suggests that one competition occurs between the anion groups of the protein present in the venom and the TPP for the cationic groups of chitosan [35].

Infections caused by different microorganisms are a threat to public health, contributing to the deaths of around 4.95 million individuals worldwide in 2019 [42]. This global threat with an estimated economic impact of US$1 billion by 2050 indicates the broad need for studies which research new antimicrobial agents that complement existing therapies [43,44]. Different nanoparticulate systems have shown antibacterial properties, with their ability to promote the cell membrane rupture of microorganisms, induce generation of reactive oxygen species or interact with intracellular components of the pathogen mainly being reported [45].

In this study, the crude venom of *T. stigmurus* exhibited no antimicrobial effect on the tested strains (Table 2 and Table 3). However, the Tsv/CN revealed an inhibitory effect on the Gram-positive strain and yeasts, showing more efficient action than the empty nanoparticles, which suggests a possible synergistic effect of the chitosan nanosystem with components present in the venom potentiating the antibiotic action. A similar effect has been reported by Torres-Rêgo et al. (2019) [36] in chitosan nanoparticles adsorbed with the TistH peptide, improving its antifungal activity. The absence of the antibacterial effect of the chitosan nanosystem adsorbed or not with Tsv on *E. coli* (Gram-negative bacteria), with antibiotic action on *S. aureus* (Gram-positive bacteria), may be associated with the different cell membrane compositions of these microorganisms, allowing a distinct molecular interaction profile [46]. Thus, studies will need to be conducted to characterize the interaction of the obtained nanosystem with components present in the membrane of these infectious agents. The conjugation of molecules with chitosan, as well as the use of nanoparticulate systems, has demonstrated the ability to increase its biological activity [45,47,48,49]. An approach developed by Auwal et al. [50] incorporated an antihypertensive biopeptide obtained from stone fish (*Actinopyga lecanora*) in cross-liked chitosan nanoparticles, finding that its hypotensive effect was improved, making the use of this chitosan nanosystem a promising approach to amplify the therapeutic efficacy of molecules.

Therefore, this approach efficiently obtained *T. stigmurus*-venom-loaded cross-linked chitosan nanoparticles which showed an ability to promote an increase in the antimicrobial efficiency of the system and antibiotic action against both bacteria and yeasts. Taken together, the data indicate the potential use of scorpion venom or its components adsorbed to chitosan nanoparticles as a useful tool for obtaining new anti-infectious agents.

## 4. Materials and Methods

### 4.1. Obtaining of Tityus stigmurus Scorpion Venom

The *Tityus stigmurus* scorpions were collected in Natal city, Rio Grande do Norte, Brazil. The scorpions were housed in Enzymology Laboratory of Federal University of Rio Grande do Norte (UFRN) in standard polypropylene cages, maintained under controlled temperature (25 ± 2 °C) with water provided ad libitum and every 15 days. They were fed cockroaches or crickets. An electrical stimulation (70 V) of the telson was induced to stimulate venom release. The samples were dissolved in ultrapure water and centrifuged at 10,000 g at 4 °C for 10 min. The supernatant was frozen (−20 °C), lyophilized (Liobrass, model L 202, São Paulo, Brazil), dissolved in phosphate-buffered saline (PBS) and the amount of venom was expressed by protein content, determined by the BCA Protein Assay kit [51]. The capture, maintenance in captivity, collection of scorpions and venom extraction were authorized by ICMBio (Chico Mendes Institute for Biodiversity Conservation, protocol number 34113 (SISBIO)). The scientific use of the material was approved by the Brazilian Genetic Heritage Management Council (SISGEN) (Process ABB2A47).

### 4.2. Preparation of T. stigmurus-Venom-Loaded Cross-Linked Chitosan Nanoparticles

The Tsv/CN was obtained for the adsorption technique and prepared using the ionic gelation method as previously described by Rocha Soares et al. (2012) with slight modifications. First, 0.1% (*w/v*) of chitosan (85% deacetylated, molecular weight: 90–190 KDa, Sigma-Aldrich^®^, St. Louis, MI, United States of America) in a 0.175% (*w*/*v*) acetic acid solution was prepared and filtered (membrane 0.45 μm), with a total volume of 5 mL for each sample. Then, 2 mL of 0.1% (*w/v*) of TPP (Sigma-Aldrich^®^, Saint Louis, MI, USA) in distilled water was added dropwise into chitosan solution under magnetic stirring of 300 rpm at temperature of 25 °C for 30 min. Spontaneous formation of opalescent dispersion was observed during synthesis. The parameters of solvents, stoichiometric ratio among compounds, pH, molecular mass and deacetylation grade of polymer were defined in previous approaches [35]. Next, the Tsv was diluted in ultrapure water to obtain a solution with a concentration of 1 mg/mL, and then aliquots of this solution were added to the chitosan nanoparticles (opalescent suspension) under magnetic stirring of 300 rpm at 25 °C for 60 min to obtain the final venom concentrations of 0.5 and 1.0% [35].

### 4.3. Physical–Chemical Aspects of Nanoparticles

#### 4.3.1. Determination of Zeta Potential, Particle Size and Polydispersity Index

The zeta potential of the particles was determined by a Doppler anemometry laser (ZetaSizer NanoZS, Malver, 82, Brookhaven, UK), applying a field strength of about 5.9 V.cm^−1^. Particle size and polydispersity index (PdI) of nanoparticles were determined using the method according to the dynamic light scattering (DLS) intensity in a particle size analyzer at 659 nm with a 173° detection angle at 25 °C. All analyses were carried out in triplicate and data expressed as mean ± standard deviation.

#### 4.3.2. Venom-Loading Efficiency

The CN, Tsv/CN 0.5% and Tsv/CN 1.0% samples were centrifuged at 20,000× *g*, 4 °C for 30 min. The supernatant venom concentrations in the samples were estimated using the BCA Protein Assay kit [51]. All analyses were performed in triplicate, and the data expressed as mean ± standard deviation (SD). The protein incorporation efficiency (IE%) was calculated using Equation (1) [41].

(1) $$\mathrm{IE}\;(\%)=\frac{\left(total\;protein-protein\;determined\;in\;the\;supernatant\right)}{total\;protein}\;\times \;100$$

#### 4.3.3. Nanoparticle Tracking Analyzer

Nanoparticle dispersion was analyzed by Nanoparticle Tracking Analyzer-NTA instrument (NANOSIGHT NS300, Malvern Panalytical, Malvern, UK) equipped with a blue 488 laser, a high-resolution sCMOS camera and a thermal detector. The NTA captures several frames of the solution and then correlates the particle size with particle concentration. After sensitivity optimization of the instrument at room temperature, a diluted sample was introduced into the measuring cell using a normal syringe. The detection, captured video, and 2D graph of concentration versus particle size were obtained and subsequently analyzed using the Stokes–Einstein relation.

#### 4.3.4. Fourier Transform Infrared Spectroscopy (FT-IR)

Fourier transform infrared spectroscopy (FT-IR) spectra analyses of CN, Tsv/CN 0.5% and Tsv/CN 1.0% were performed by a Prestige 21 FT-IR spectrophotometer (Shimadzu, Tokyo, Japan) at 25 °C, in the scanning range from 4000 to 400 cm^−1^, according to the methodology described by Soares et al. [38]. The CN, Tsv, Tsv/CN 0.5%, Tsv/CN 1.0% and pure compounds were subsequently dried in a speed vacuum concentrator (Centrivap; Labconco, Kansas City, United States of America). The samples were mixed with potassium bromide (KBr) in weight ratio between the powder and KBr 1:200 *w*/*w* in a melting pot and then compressed in a hydraulic press.

#### 4.3.5. Study of Shape and Surface of Nanoparticles

The shape and surface aspect of CN, Tsv/CN 0.5% and Tsv/CN 1.0% were evaluated by field emission gun scanning electronic microscopy (FEGSEM, Zeiss Microscopy, Auriga, Jena, Germany). The samples were prepared for the FEGSEM images by applying one drop of the nanoparticle dispersion on a carbon tape slide and dried under a desiccator for 24 h until analysis.

### 4.4. Evaluation of the In Vitro Antibacterial Activity

Bacterial strains were obtained from a Clinical Microbiology Laboratory at UFRN and maintained in nutrient agar (HIMEDIA^®^, Mumbai, Maharashtra, India) at 4 °C, or stored in beads at −20 °C. Gram-positive and Gram-negative *Staphylococcus aureus* (ATCC 29213) and *Escherichia coli* (ATCC 25922) bacteria were used for the antimicrobial assays. The antimicrobial activity of the Tsv/CN were evaluated by determining the MICs using the broth microdilution method in Mueller–Hinton broth (MHB), as described in the Clinical and Laboratory Standards Institute (CLSI). The inoculum was prepared at 1 × 10^6^ colony forming unit per milliliter (CFU/mL). First, cells were grown overnight at 35 °C, and 50 µL of the microbe suspension was subsequently added to serial dilutions of the Tsv, CN, Tsv/CN 0.5% and Tsv/CN 1.0% (assay total volume corresponded to 100 µL). The assay was done in a 96-well microplate and the samples were incubated at 35 ± 2 °C, then agitated at 200 rpm for 24 h, after which the optical density was measured at 595 nm using a microplate reader (Epoch Biotek, Winooski, United States of America). Wells containing MHB and 0.9% saline solution were used as sterility control and the bacterial suspension in the absence of nanoparticles was used as positive control.

### 4.5. Evaluation of the In Vitro Antifungal Activity

The strains were obtained from the mycological culture collection belonging to the Laboratory of Medical and Molecular Mycology, Department of Clinical and Toxicological Analyses, Federal University of Rio Grande do Norte, Brazil. In vitro reference strains of *Candida* spp. provided by the American Type Culture Collection (ATCC) and the Netherlands’ collection at the Centraalbureau voor Schimmelcultures (CBS) were used to analyze the antifungal activity, as follows: *C. albicans* ATCC90028, *C. tropicalis* ATCC13803, *C. dubliniensis* CBS7987, *C. glabrata* ATCC2001, *C. parapsilosis* ATCC22019, *C. metapsilosis* ATCC 96143, *C. krusei* ATCC6258 and *C. rugosa* ATCC 10571. The strains were stored at −80 °C in yeast extract peptone dextrose (YPD) broth (10 g/L yeast extract, 20 g/L glucose, and 20 g/L mycological peptone) containing 20% (*v*/*v*) glycerol. In order to determine minimal inhibitory concentrations (MICs), *Candida* cell inoculum of all the tested strains were obtained after 48 h of cultivation on Sabouraud Dextrose Agar (SDA) at 35 °C and an initial cellular suspension was prepared according to the McFarland scale 0.5 standard (1 to 5 × 10^6^ cells). Next, two serial dilutions were made, the first one in saline solution (1:50) and the second one in Mueller–Hinton broth medium (Difco) (1:30). Aliquots of 100 μL of the samples were then transferred to microtiter 96-well plates containing 100 μL of serial dilution concentrations of Tsv/CN.

Another experiment was performed with cells grown in the absence of the venom (Mueller–Hinton broth only) and Tsv. Finally, the plates were incubated at 37 °C and test readings taken after 48 h of incubation. MIC 50% was considered the lowest concentration of samples capable of inhibiting 50% of the growth of each strain, taking the respective positive growth control as reference (treated in the same manner, but without the nanoparticle added to the yeast cells). MIC 100% was considered the lowest concentration of nanoparticles capable of inhibiting any visual growth of each strain. The assay was performed according to the M27-A3 protocol recommended by the Clinical Laboratory Standards Institute (CLSI) adapted for non-commercial antifungal drugs.

## 5. Conclusions

The Tsv/CN obtained by the ionic gelation technique revealed the high encapsulation efficiency of the venom components, resulting in a homogeneous cationic dispersion of reduced size. The Tsv/CN also revealed broad antimicrobial activity against species of high medical importance, acting against Gram-positive bacteria and different strains of the *Candida* genus. The greater antimicrobial action of Tsv/CN when compared to the venom or the nanosystem alone indicates the beneficial effect of the combination, enhancing the antibiotic action. Therefore, the data from this approach shed light on the use of the nanosystem associated with venom components as a promising approach for developing new anti-infective agents.

## Figures and Tables

**Table 1:** Particle size, polydispersity index (PdI), zeta potential and encapsulation efficiency of chitosan nanoparticles (CN) and *Tityus stigmurus*-venom-loaded cross-linked chitosan nanoparticles at 0.5% (Tsv/CN 0.5%) and 1.0% (Tsv/CN 1.0%).

Samples	Size (nm)	PdI	Zeta Potential (mV)	Encapsulation Efficiency (%)
CN	134.40 ± 0.75	0.391 ± 0.068	+23.20 ± 1.47	---
Tsv/CN 0.5%	176.16 ± 1.45	0.465 ± 0.027	+28.63 ± 0.58	81.36
Tsv/CN 1.0%	106.03 ± 1.94	0.430 ± 0.014	+26.96 ± 0.58	78.67

Values are mean ± standard deviation (S.D.), *n* = 3.

**Table 2:** Determination of MICs of *Tityus stigmurus* venom, chitosan nanoparticles and *Tityus stigmurus*-venom-loaded cross-linked chitosan nanoparticles at concentrations of 0.5% and 1.0% against bacterial strains.

Strains	Tsv (µg/mL)	CN (µg/mL)	Tsv/CN 0.5% (µg/mL)	Tsv/CN 1.0% (µg/mL)
	MIC 100%	MIC 100%	MIC 100%	MIC 100%
*Staphylococcus aureus* (ATCC 29213)	>500	178.5	89.2	44.6
*Escherichia coli* (ATCC 25922)	>500	178.5	>178.5	>178.5

MIC (Minimum inhibitory concentration); Tsv *(Tityus stigmurus* venom); CN (chitosan nanoparticles); Tsv/CN 0.5% (*T. stigmurus*-venom-loaded cross-linked chitosan nanoparticles at 0.5% concentration) and Tsv/CN 1.0% (*T. stigmurus*-venom-loaded cross-linked chitosan nanoparticles at 1.0% concentration). Each experiment was performed in duplicate and each sample in quadruplicate.

**Table 3:** Determination of antifungal activity of *Tityus stigmurus* venom, chitosan nanoparticles and *Tityus stigmurus*-venom-loaded cross-linked chitosan nanoparticles at concentrations of 0.5% and 1.0% against *Candida* spp.

Strains	Tsv (µg/mL)	CN (µg/mL)	Tsv/CN 0.5% (µg/mL)	Tsv/CN 1.0% (µg/mL)
	MIC 100%	MIC 100%	MIC 100%	MIC 100%
*C. albicans* (ATCC 90028)	>178.5	178.5	178.5	178.5
*C. tropicalis* (ATCC 13803)	>178.5	11.1	11.1	5.5
*C. dubliniensis* (CBS 7987)	>178.5	178.5	178.5	178.5
*C. glabrata* (ATCC 2001)	>178.5	>178.5	>178.5	>178.5
*C. parapsilosis* (ATCC 22019)	>178.5	22.2	22.2	11.1
*C. krusei* (ATCC 6258)	>178.5	>178.5	>178.5	>178.5
*C. rugosa* (ATCC 10571)	>178.5	178.5	178.5	178.5

MIC (Minimum inhibitory concentration); Tsv *(Tityus stigmurus* venom); CN (chitosan nanoparticles); Tsv/CN 0.5% (*T. stigmurus*-venom-loaded cross-linked chitosan nanoparticles at 0.5% concentration) and Tsv/CN 1.0% (*T. stigmurus*-venom-loaded cross-linked chitosan nanoparticles at 1.0% concentration). Each experiment was performed in duplicate and each sample in quadruplicate.

## Data Availability

Data are contained within the article.
